# ^1^H NMR Study of the Enantioselective Binding of λ- and Δ-[Ru(bpy)_2_(m-bpy-GHK)]Cl_2_ to the Deoxynucleotide Duplex d(5'-C_1_G_2_C_3_G_4_A_5_A_6_T_7_T_8_C_9_G_10_C_11_G_12_-3')_2_

**DOI:** 10.1155/BCA.2005.109

**Published:** 2005

**Authors:** A. Myari, N. Hadjiliadis, A. Garoufis

**Affiliations:** Laboratory of Inorganic and General Chemistry Department of Chemistry University of Ioannnina Ioannina 45110 Greece

## Abstract

The interaction of the diastereomeric complexes Λ- and Δ - [Ru(bpy)_2_(m - GHK)]Cl_2_, (GHK = glycine-histidine-lysine) to the deoxynucleotide duplex d(5'-CGCGAATTCGCG-3')_2_ was studied by means of ^1^H NMR spectroscopy. The diastereomers interact with the oligonucleotide duplex differently. The Δ - [Ru(bpy)_2_ (m - GHK)]Cl_2_ is characterized by major groove binding close to the central part of the oligonucleotide, with both the peptide and the bipyridine ligand of the complex involved in the binding. The λ - [Ru(bpy)_2_ (m - bpy - GHK)]C_2_ binds loosely, approaching the helix from the minor groove. The NMR analysis shows that the peptide (GHK) binding has a determinative role in the interactions of both diastereomers with the oligonucleotide.


### $^1$H NMR Study of the Enantioselective Binding of Λ- and Δ-[Ru(bpy)$_2$(m-bpy-GHK)]Cl$_2$ to the Deoxynucleotide Duplex d(5'-$C_1G_2C_3G_4A_5A_6T_7T_8C_9G_{10}C_{11}G_{12}$-3')$_2$

A. Myari, N. Hadjiliadis*, A. Garoufis*

*Laboratory of Inorganic and General Chemistry, Department of Chemistry, University of Ioannnina, Ioannina 45110, Greece.*

### ABSTRACT

The interaction of the diastereomeric complexes Λ- and Δ-[Ru(bpy)$_2$(m-GHK)]Cl$_2$, (GHK = glycine-histidine-lysine) to the deoxynucleotide duplex d(5'-CGCGAATTCGCG-3')$_2$ was studied by means of $^1$H NMR spectroscopy. The diastereomers interact with the oligonucleotide duplex differently. The Δ-[Ru(bpy)$_2$(m-GHK)]Cl$_2$ is characterized by major groove binding close to the central part of the oligonucleotide, with both the peptide and the bipyridine ligand of the complex involved in the binding. The Λ-[Ru(bpy)$_2$(m-bpy-GHK)]Cl$_2$ binds loosely, approaching the helix from the minor groove. The NMR analysis shows that the peptide (GHK) binding has a determinative role in the interactions of both diastereomers with the oligonucleotide.

### 1. INTRODUCTION

Over the last decades, DNA-binding low molecular weight compounds have found considerable application as chemotherapeutic agents. On a molecular basis, their cytotoxic effect originates from their interaction with the DNA double helix, often in a non-covalent way. This type of reversible interaction takes place in three primary ways /1/: (i) surface binding which is generally non-specific and primarily electrostatic in origin e.g. in the case of multiple charged simple cations such as magnesium and cations of simple organic amines /1/, (ii) groove binding interactions e.g. netropsin /2/, distamycin /3/, Hoechst 33258 /4/ and SN 6999 /5/, and (iii) intercalation of a planar or approximately planar aromatic ring system between base pairs as in the case of ethidium /6/, adriamycin /7/ and daunomycin /8/.

There is increasing interest in the chemical design of small molecular mimics of DNA-binding proteins

* Corresponding authors.

NH: Tel +30 651 98420 , e-mail nhadjil@cc.uoi.gr Fax +3 0651 44831

AG: Tel +30 651 98409 , e-mail agaroufi@cc.uoi.gr Fax +3 0651 44831

playing an important regulatory role in controlling replication and transcription of genomic material. The sequence-specific binding of such molecules to DNA might affect replication, transcription or other physiological functions of the cell. The extremely high specificity upon recognition and binding to DNA target sites, of protein molecules such as restriction endonucleases, provides a very good basis for the design of sequence-specific DNA binders. The recognition process of the cognate DNA fragment by these proteins often involves base-specific interactions between the DNA bases and a recognition loop of amino acid residues, comprising mainly of hydrogen bonding schemes and Van der Waals interactions /9-11/.

Despite their substantial contribution to sequence specificity, individual recognition peptide sequences lack the ability to bind tightly to DNA, a property sufficiently provided by the non-specific contacts made by the protein backbone /12-14/. On the other hand, positively-charged metal complexes can associate within the grooves of polyanionic DNA, with the binding being further stabilized by a variety of intermolecular forces such as Van der Waals, hydrophobic interactions and hydrogen bonding. Footprinting studies have shown that site-specific recognition by conjugation of small peptides to metal complexes can be successful /15-16/. Therefore, on the basis of the known high affinity of ruthenium polypyridine complexes towards DNA binding /17-19/, $[Ru(bpy)_3]^{2+}$ was chosen for conjugation with the recognition peptide sequence.

As a starting peptide sequence, Gly-His-Lys (GHK), the growth modulating factor was chosen. Its water solubility as well as its capacity to facilitate transportation within the cell comprise essential characteristics for a DNA-targeted molecule. Moreover, its Cu(II) complex is known to adopt a specific orientation when interacting with the minor groove of DNA, thus introducing specificity in DNA binding /20/.

The DNA binding properties of our designed complex were tested on the spectroscopically and crystallographically well-characterized Dickerson-Drew dodecamer, $d(CGCGAATTCGCG)_2$ which forms a self-complementary duplex whose structure is understood in detail /21/.

### 2. EXPERIMENTAL

The measurements were made on a Varian Unity-500 MHz instrument. 1D-$^{1}$HNMR spectra were recorded at 303 K into 4096 data points, with a 6024 Hz spectral width after 128 transients. $^{1}$H NOESY spectra were recorded in phase sensitive mode with a total 2048 X 256 points for mixing time 200 to 400 ms, while $^{1}$H ROESY spectra at mixing times ($\tau_m$) 60 –120 ms. $^{1}$H DQF COSY spectra were collected using TPPI method, in a spectral width of 3125 Hz with total 2048 X 256 points and a relaxation delay of 1.5 s.

The amounts of the oligonucleotide were estimated by weighing and the concentration of the sample was determined using its absorption at 260 nm. In all NMR experiments carried out, a 100 mM $Na_2HPO_4/NaH_2PO_4$ ($p$H = 7.00) buffer was used. The lyophilized samples were dissolved in $D_2O$ (99.96) and lyophilized again to dryness.

The 1D $^{1}$H NMR spectra were recorded on sample concentration ~100 $OD_{260}$ units while the 2D NOE experiments in more concentrated samples (~ 300 $OD_{260}$). $^{1}$H NMR spectra of the labile oligonucleotide protons were recorded in 90% $H_2O$ 10% $D_2O$ (field-frequency lock). No internal chemical shift reference was added to the samples.

### 3. RESULTS AND DISCUSSION

### 2.1. Synthesis and characterization of Λ- and Δ-[Ru(bpy)$_2$(m-GHK)]Cl$_2$ enantiomers, (bpy=2,2'-bipyridine, m-GHK=4-methyl-4'-glycyl-histidyl-lysyl-2,2'-bipyridine).

The synthesis of the diastereomers Λ- and Δ-[Ru(bpy)$_2$(m-bpy-GHK)]Cl$_2$ was based on the enantiomerically pure complexes Λ- and Δ-*cis*-[Ru(bpy)$_2$(py)$_2$]$^{2+}$ as building blocks. Details of the synthesis will be described elsewhere /22/. The structures of Δ- and Λ-[Ru(bpy)$_2$(m-bpy-GHK)]$^{2+}$ are shown in figure 1. Both complexes were characterized by elemental analysis, ESI-MS and $^1$H-NMR spectroscopy. The enantiomeric purity of the ruthenium complexes was checked by CD spectroscopy /22/.

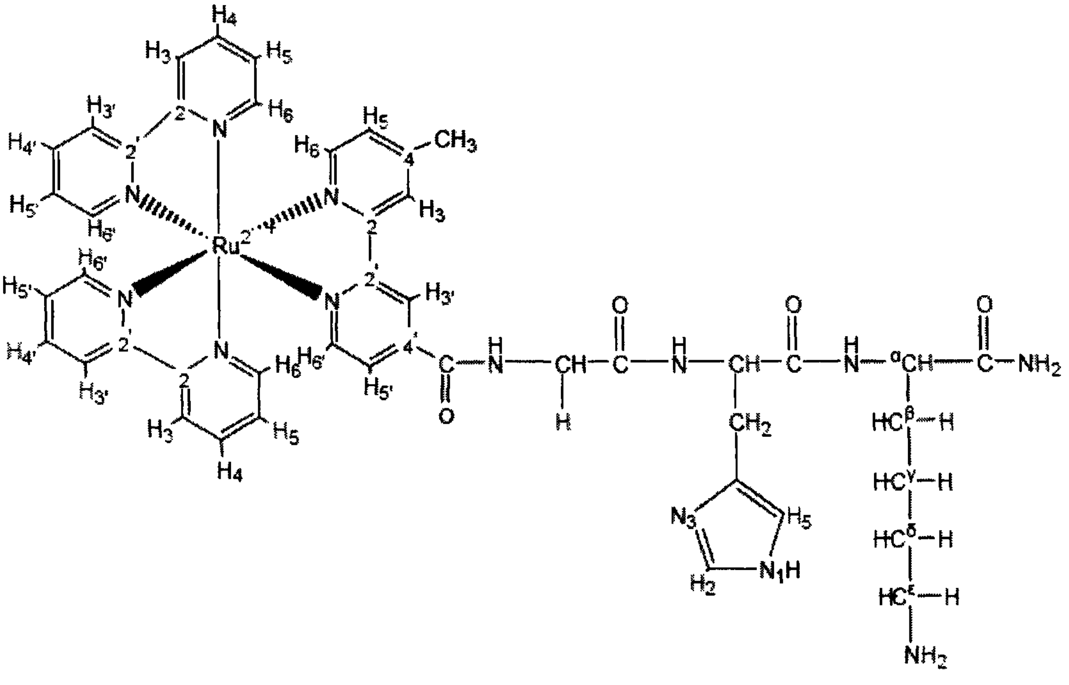

**Λ-[Ru(bpy)$_2$(m-GHK)]$^{2+}$**

**Fig. 1:** Structure and atom numbering of Λ-[Ru(bpy)$_2$(m-GHK)]$^{2+}$

### 2.2. Interactions of the [Ru(bpy)$_2$(m-GHK)]Cl$_2$ enantiomers with the DNA dodecamer duplex d(5'-CGCGAATTCGCG-3')$_2$.

The binding of the two diastereomeric complexes Δ- and Λ [Ru(bpy)$_2$(m-GHK)]$^{2+}$ to the DNA dodecamer duplex d(5'-CGCGAATTCGCG-3')$_2$ has been studied by the DQF COSY techniques and two dimensional NOE spectroscopy. The 2D NMR studies reveal different binding modes for the two diastereomers.

As seen from the 1D $^1$H NMR spectra of the samples containing Δ- or Λ-diastereomer : oligonucleotide 1:1 (0.1 M phosphate buffer, pH=7.0, T=298 °K), only one set of resonances was observed indicating that the complex is in fast exchange binding kinetics (Figure 2).

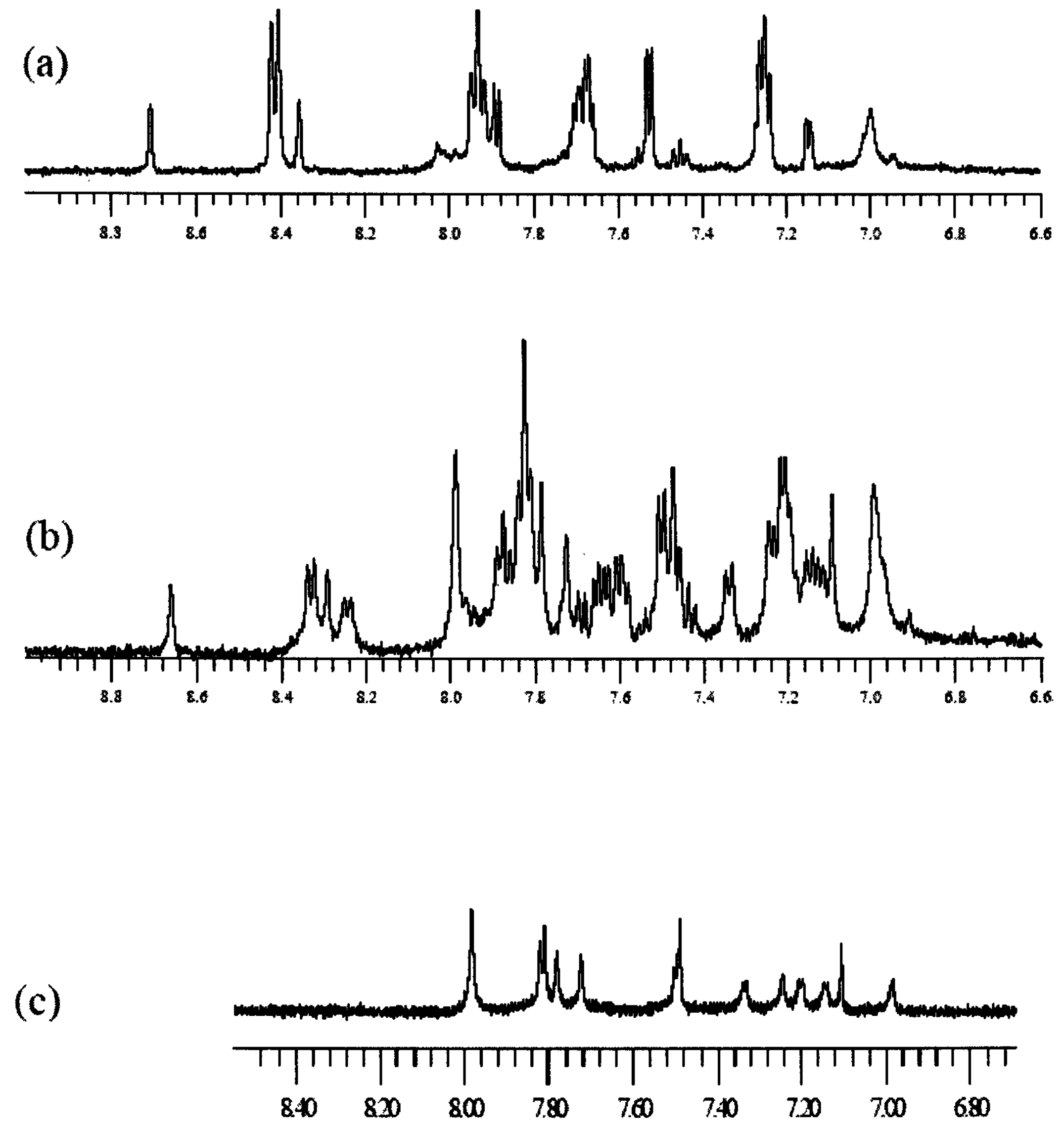

**Fig. 2:** $^1$H NMR spectra of the aromatic region (a) of the free Δ-$[Ru(bpy)_2(m\text{-}bpy)\text{-}GHK)]^{2+}$ (b) with the added dodecanucleotide at ratio 1:1, (c) the free dodecanucleotide.

Upfield shifts of the bpy proton signals of both enantiomers were observed, ranging from 0.039-0.192 ppm for the Δ-isomer and 0.026-0.066 for the Λ-isomer. Moreover, the aromatic protons of m-GHK ligand show small upfield shifts (less than 0.1 ppm) for both enantiomers. These values are not indicative of ligand binding to the oligonucleotide by intercalation. In general, intercalation causes large upfield changes in the resonances of the ligand protons (0.3-1.0 ppm) and significant broadening of the signals due to intermediate exchange /23/. The observed electron shielding of H3 (0.192 ppm for Δ-isomer) and H4 (0.168 ppm for Δ-isomer) bipyridyl protons due to the interaction with the DNA bases is close to the lower limit for intercalation, implying that the vertical distance between these protons and the base planes is not very high /24,25/. On the other hand, the upfield shifts of bpy protons observed for the Λ-isomer (less than 0.1 ppm) suggest very weak association with the oligonucleotide.

The participation of the side chain amino group of lysine in DNA binding is evidenced for both isomers by the upfield shift of the $H_\varepsilon$ protons (0.09 ppm for Δ- and 0.074 ppm for Λ-isomer). The binding of the positive charge amino group of lysine has also been observed in the case of Lys-Tyr-Lys /26/ and Lys-Trp-Lys /27/ where the binding to DNA takes places *via* electrostatic interactions between the lysine residues and the polyanionic DNA backbone. Significant interaction of the imidazole H2 protons of histidine is observed for the Λ-isomer (0.118 ppm downfield shift). Furthermore upfield shift (0.13 ppm) of the exchangeable NH protons of His was observed for both enantiomers suggesting the participation of the peptide bond in DNA binding.

A number of NOESY cross-peaks between protons of the metal complex and protons of the oligonucleotide were observed indicating interproton distances less than 5 Å (Figures 3 and 4). In the case of Δ-[Ru(bpy)$_2$(m-GHK)]Cl$_2$, distances of less than 5Å were observed between the aromatic protons of m-bpy ligand and the H2" protons of the A5, A6 and C9 bases (in the complementary strand) all facing the major groove of the helix. The NH protons of the peptide backbone show cross-peaks with protons that are also accessible from the major groove. Intermolecular NOE's between the lysine aliphatic side chain and the oligonucleotide protons indicate that this part of the peptide is located close to the helix.

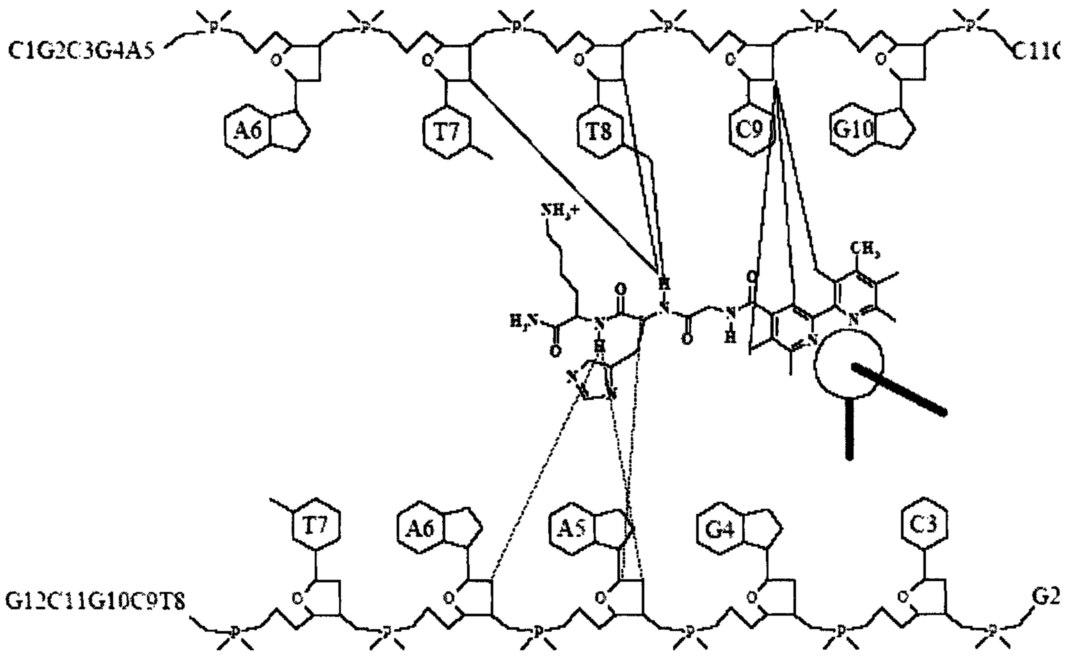

**Fig. 3:** Model of observed intermolecular NOEs between the Δ-[Ru(bpy)$_2$(m-bpy)-GHK)]$^{2+}$ and the dodecanucleotide.

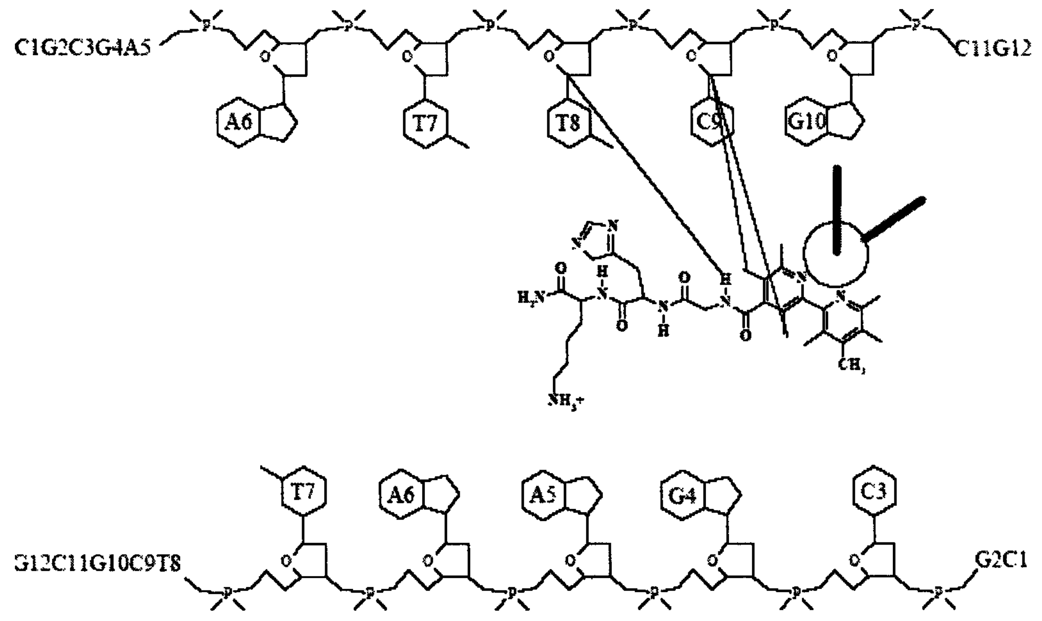

**Fig. 4:** Model of observed intermolecular NOEs between the Λ $[Ru(bpy)_2(m\text{-}GHK)]^{2+}$ and the dodecanucleotide.

On the other hand, the intermolecular NOE contacts between the Λ-$[Ru(bpy)_2(m\text{-}GHK)]Cl_2$ and the oligonucleotide are significantly less compared to the Δ- enantiomer suggesting a looser binding of the former. A few of these take place between the ligand m-bpy protons H3', H5' and the A6H1'and C9H1' and between the peptide backbone Gly-NH and the sugar proton H1' of T8, all located in the helix minor groove. In contrast to the Δ- isomer, no cross-peak was observed between the other two bpy ligand protons of the complex and the oligonucleotide indicating that only the m-bpy-GHK moiety of the complex binds to the d(5'-CGCGAATTCGCG-3')$_2$.

Major and minor grooves differ significantly in electrostatic potential, hydrogen bond characteristics, steric effects and hydration. Therefore, many proteins exhibit binding specificity primarily through major groove interactions while small groove binding molecules like netropsin and distamycin generally prefer the minor groove of DNA /1/. Unlike the other non-intercalative molecules, Δ-$[Ru(bpy)_2(m\text{-}GHK)]^{2+}$ forms specific contacts with the walls of the major groove of DNA. This can be partially interpreted by the tendency of the bpy ligand to align itself close to the basepairs /28/, a fact already seen in the significant shifts of the aromatic bpy protons. This alignment is probably hindered in the case of the Λ-isomer where the bpy ligands do not participate in DNA binding, whereas the enantiomer binds from the minor groove.

In order to investigate the site-specificity of $[Ru(bpy)_2(m\text{-}GHK)]^{2+}$ in DNA binding, its interaction with d(CGCGATCGCG)$_2$, also a β-type DNA, is under study. Preliminary results indicate weaker interaction between both isomers and the decanucleotide duplex compared to the dodecanucleotide. The binding of the Δ-isomer takes place from the major groove in the region of A5/T8, A6/T7 base extended until the ends of the dodecanucleotide. The Λ-enantiomer interacts very weakly with the double helix (shifts less than 0.05 ppm), probably through electrostatic interactions between the metal complex and the oligonucleotide backbone. Complementary molecular modeling studies are currently underway.

### 3.3. Conclusion

In conclusion, the NMR experiments presented show that the $[Ru(bpy)_2(m\text{-}GHK)]^{2+}$ complex binds differently to oligonucleotides. Major groove binding to $d(CGCGAATTCGCG)_2$ was observed for Δ-$[Ru(bpy)_2(m\text{-}GHK)]Cl_2$ with the histidyl-lysyl part of the peptide ligand recognising the adjacent C9G10C11 of the oligonucleotide sequence, thus placing the bpy ligands close to the central part. The Λ-isomer approaches the double helix from the minor groove, with the aromatic protons of ligand m-GHK interacting weakly (Figure 5).

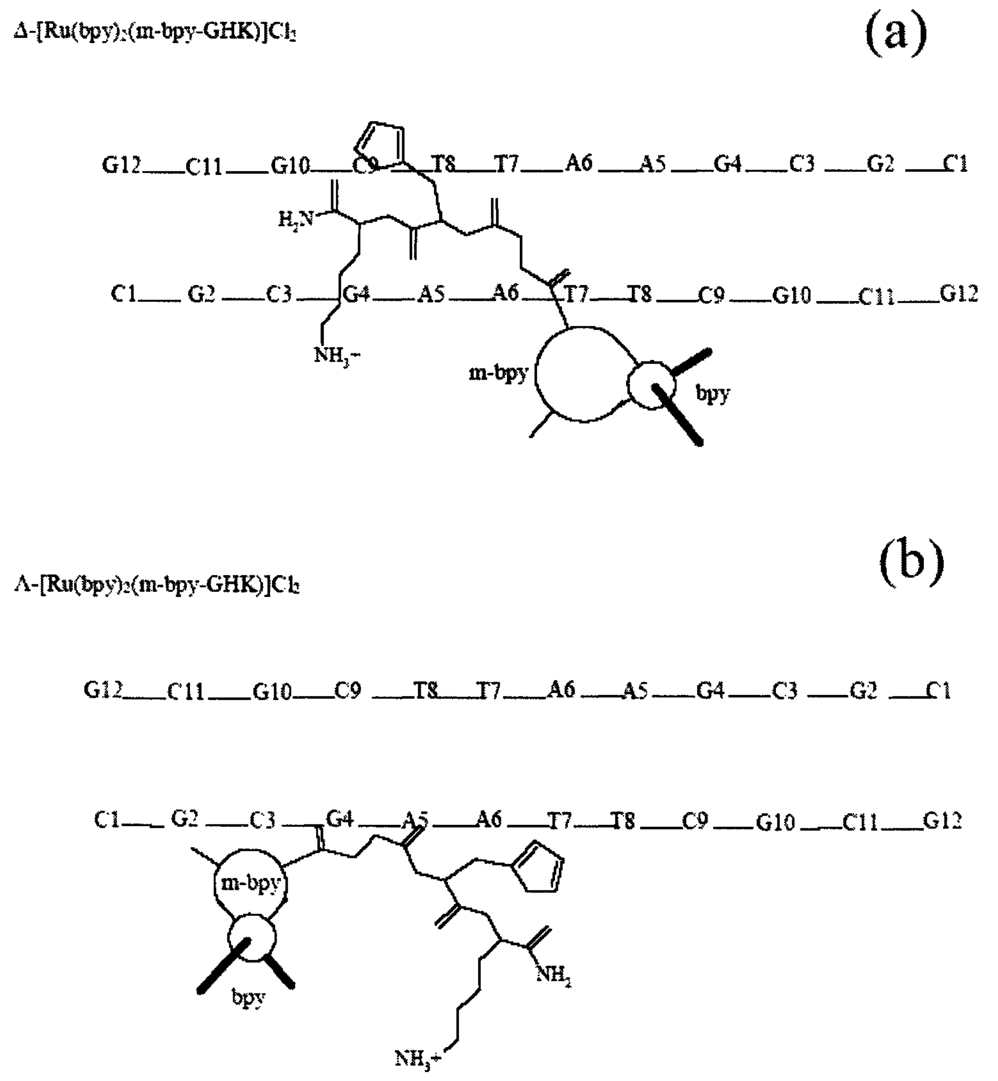

**Fig. 5:** A qualitative binding model of (a) Δ $[Ru(bpy)_2(m\text{-}bpy\text{-}GHK)]^{2+}$ and the dodecanucleotide and (b) Λ $[Ru(bpy)_2(m\text{-}bpy\text{-}GHK)]^{2+}$ and the dodecanucleotide.

The site-specificity in DNA binding of the enantiomeric ruthenium complexes is indicated by preliminary results of their interaction with the nucleotide duplex d(CGCGATCGCG)$_2$, where the change of the central part of the oligonucleotide sequence affects dramatically the ability of the complex to recognise and associate to its binding site.

### ACKNOWLEDGEMENTS

The financial support of this work by the Greek General Secretariat for Research and Technology and the European Commission within the frame of the PENED 01 projects is thankfully acknowledged (Grant No. 01GA367). A.M. would also like to thank the graduate programme in Bioinorganic Chemistry coordinated by Prof. N. Hadjiliadis.

### 4. REFERENCES

1. G.M. Blackburn and M.J. Gait (Eds) "*Nucleic Acids in Chemistry and Biology*", 2$^{nd}$ edition, Oxford University Press Inc, New York, 1996.
2. M.L. Kopka, C. Yoon, D. Goddsell, P. Pjura and R.E. Dickerson, *Proc. Nat. Acad. Sci. USA* 82, 1376-1380 (1985).
3. M. Coll, C.A. Frederick, A.H.-J. Wang and A. Rich, *Proc. Nat. Acad. Sci. USA* 84, 8385-8389 (1987).
4. M.S. Searle and K.J. Embrey, *Nucl. Acid. Res.* **18**, 3753-62 (1990).
5. W. Leupin, W.J. Chazin, S. Hyberts, W.A. Denny and K. Wuthrich, *Biochemistry*, **25**, 5902-5910 (1986).
6. W.D. Wilson, C.R. Krishnamoorthy, Y.-H. Wang and J.C. Smith, *Biopolymers*, **24**, 1941-19651 (1985).
7. C.A. Frederick, L.D. Williams, G. Ughetto, G.A. Van der Marel, J.H. Van Boom, A. Rich and A.H.J. Wang, *Biochemistry* **29**, 2538-2549 (1990).
8. A.H.-J. Wang, G. Ughetto, G.J. Quigley and A. Rich, *Biochemistry*, **26**, 1152-1163 (1987).
9. M. Newman, K. Lunnen, G. Wilson, J. Greci, I. Schildkraut and S.E.V. Phillips, *EMBO J.*, **17**, 5466-5476 (1998).
10. F.K. Winkler, D.W. Banner, C. Oefner, D. Tsernoglou, P.S. Brown, S.P. Heathman, R.K. Bryan, P.D. Martin, K. Petratos and K.W. Wilson, *EMBO J.*, **5**, 1781-1795 (1993).
11. M. Newman, T. Strzelecka, L.F. Dorner, I. Schildkraut and A.K. Aggarwal, *Science*, **269**, 656-663 (1995).
12. B. Cuenoud and A. Schepartz, *Science*, **259**, 510-513 (1993).
13. T. Morii, M. Simomura, S. Morimoto and J. Saito, *J. Am. Chem. Soc.*, **115**, 1150-1151 (1993).
14. M.F. Bruist, S.J. Horvath, L.E. Hood, T.A. Steitz and M.I. Simon, *Science*, **235**, 777-780 (1987).
15. N.Y. Sardesai, K. Zimmerman and J.K. Barton, *J. Am. Chem. Soc.*, **116**, 7502-7508 (1994).
16. N.Y. Sardesai and J.K. Barton, *J. Biol. Inorg. Chem.*, **2**, 762-771 (1997).
17. J.G. Collins, A.D. Sleeman, J.R. Aldrich-Wright, I. Greguric and T.W. Hambley, *Inorg. Chem.*, **37**,

3133-3141 (1998).

18. F.M. Foley, F.R. Keene and J.G. Collins, *J. Chem. Soc. Dalton Trans.*, **2001**, 2968-2974.
19. J.P. Rehmam and J.K. Barton, *Biochemistry*, **1990**, 1701-1709.
20. D.S. Sigman, A. Mazumder and D.M. Perrin, *Chem. Rev.*, **93**, 2295-2316 (1993).
21. (a) H.R. Drew and R.E. Dickerson, *J. Mol. Biol.*, **151**, 5353-556 (1981), (b) D.J. Patel, A. Pardi and K. Itakura, *Science* **216**, 581-590 (1982), (c) D.J. Patel, S. Ikuta, S. Kozlowski and K. Itakura, *Proc. Natl. Acad. Sci. U.S.A.*, **80**, 2184-2188 (1983), (d) D.R. Hare, D.E. Wemmer, S. Chou, G.J. Drobny and B.R. Reid, *J. Mol. Biol.*, **171**, 319-336 (1983), (e) X. Shui, C.C. Sines, L. McFail-Isom, D. Van Derveer and L.D. Williams, *Biochemistry*, **37**, 16877-16887 (1998), (f) V. Tereshko, G. Minasov and M. Egli, *J. Am. Chem. Soc.*, **121**, 470-471 (1999).
22. A. Myari, N. Hadjiliadis and A. Garoufis, unpublished data.
23. C.M. Dupureur and J.K. Barton, *Inorg. Chem.* **36**, 33-38 (1997).
24. C. Giessner-Prettre and B. Pulman, *Acad. Sci. Paris Ser D.* **283**, 675-676 (1976).
25. C. Giessner-Prettre, B. Pulman, P.N. Borer, L.S/ Kan and P.O.P. Ts'o, *Biopolymers* **15**, 2277-2286 (1976).
26. R. Barthwal, A. Mujeeb, S. Kukreti, A. Gupta and G. Govil, *J. Mol. Recogn.* **4**, 45-52 (1991).
27. F. Brun, J.J. Toulme and C. Helene, *Biochemistry* **14**, 558-563 (1975).
28. P. Lincoln and B. Norden, *J. Phys. Chem. B* **102**, 9583-9594 (1998).

